# Impact of Lipophilicity‐Tuning on the Antimicrobial Activity of a Series of *β*‐Face–Expanding Bile Acid Derivatives

**DOI:** 10.1155/bmri/5550616

**Published:** 2026-07-30

**Authors:** Allan Mora Abarca, Luis Rivera-Montero, Kenia Barrantes, Victor H. Soto-Tellini, William J. Zamora

**Affiliations:** ^1^ Center for Research in Electrochemistry and Chemical Energy (CELEQ), University of Costa Rica, San José, Costa Rica, ucr.ac.cr; ^2^ Health Research Institute, University of Costa Rica, San José, Costa Rica, ucr.ac.cr; ^3^ CBio3 Laboratory, School of Chemistry, University of Costa Rica, San José, Costa Rica, ucr.ac.cr; ^4^ Laboratory of Computational Toxicology and Artificial Intelligence (LaToxCIA), Biological Testing Laboratory (LEBi), University of Costa Rica, San José, Costa Rica, ucr.ac.cr; ^5^ National Advanced Computing Collaboratory (CNCA), National High Technology Center (CeNAT), San José, Costa Rica

**Keywords:** antimicrobial activity, *E. faecalis*, lipophilicity, *S. aureus*, steroid derivatives

## Abstract

The increasing prevalence of antimicrobial resistance has stimulated the search for new molecular scaffolds with improved efficacy and selectivity. In this study, five bile acid–derived aminosteroids (AC series) and their corresponding biphenyl‐functionalized derivatives (BIAC series) were synthesized and evaluated against *Staphylococcus aureus* and *Enterococcus faecalis*. Antimicrobial activity, hemolytic activity, and lipophilicity were investigated to establish structure–activity relationships. Quantum mechanics–derived lipophilicity (QM‐log*P*) successfully captured subtle differences associated with the number, position, and orientation of hydroxyl groups on the steroid nucleus, providing a level of discrimination not achieved by conventional fragment‐based methods. Introduction of the biphenyl moiety significantly increased lipophilicity and resulted in enhanced antimicrobial activity throughout the BIAC series compared with the parent AC compounds. BIAC05Q emerged as the most promising derivative, combining potent antibacterial activity with low hemolysis. The superior performance of BIAC05Q appears to be associated with the presence of an axial hydroxyl group at C7, which promotes a favorable amphiphilic balance and aggregation propensity. Collectively, the results suggest that the antimicrobial activity of these bile acid derivatives is closely linked to their supramolecular behavior and support the hypothesis that self‐assembled aggregates, rather than individual molecules, may constitute the bioactive species. These findings highlight the value of QM‐log*P*–guided molecular design as a strategy to optimize amphiphilicity, aggregation, antimicrobial activity, and selectivity in bile acid–based antimicrobial agents.

## 1. Introduction

The incidence of nosocomial infections associated with antibiotic‐resistant bacteria and biofilm formation continues to rise worldwide [1]. *Staphylococcus aureus* and *Enterococcus faecalis* are among the most important gram‐positive pathogens responsible for healthcare‐associated (nosocomial) infections worldwide. *S. aureus* is a leading cause of bloodstream infections, surgical site infections, ventilator‐associated pneumonia, infective endocarditis, and device‐related infections [2], whereas *E. faecalis*is frequently associated with urinary tract infections, bacteremia, endocarditis, and infections linked to indwelling medical devices [3]. The clinical significance of these pathogens is further amplified by the emergence and dissemination of multidrug‐resistant strains, including methicillin‐resistant *Staphylococcus aureus* (MRSA) and vancomycin‐resistant enterococci (VRE), which contribute to increased morbidity, mortality, length of hospitalization, and healthcare costs. These situations highlight the need for rational molecular design of new, tunable antimicrobial agents that can reduce bacterial loads via an accessible synthetic pathway [4]. In this context, amphiphilic molecules, which possess both lipophilic and hydrophilic groups, appear to be promising scaffolds for antimicrobial agents, as they can be tailored with ionic residues that interact with the negatively charged membrane, whereas lipophilic residues disrupt the cell membrane [5–8]. However, excessive lipophilicity can promote nonspecific interactions with membranes and increase cytotoxic effects [9]. Therefore, identifying fragments with optimal lipophilicity is crucial in the rational design of antimicrobial agents, ranging from therapeutic antibiotics to disinfectants intended for safe human use.

**Figure 1:**
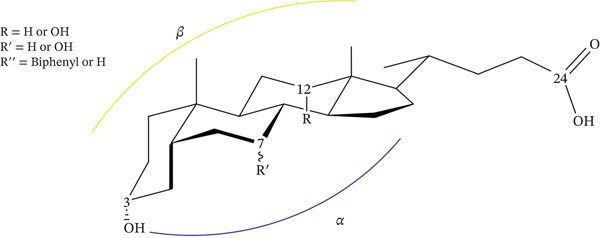
Molecular structure of bile acid, showing the lipophilic face (or *β*‐face) with lipophilic groups (methyl) and the hydrophilic face (or *α*‐face) with hydrophilic groups (hydroxyl′s).

Natural amphiphilic substance is represented by bile acids, which are derived from cholesterol metabolism. Figure 1 shows a unique substitution pattern of these molecules [4] with an amphiphilic bifacial structure, a *β*‐face (the hydrophobic one, which contains methyl groups) and *α*‐face (the hydrophilic one, which features hydroxyl groups at Positions 3, 7, and 12 of the steroid skeleton), as well as an acid group at Position 24. This unique structural configuration provides a versatile framework for developing novel derivatives throughout an easy synthetic pathway [10–12]. Moreover, their intrinsic lipophilicity [13], allows them to aggregate in aqueous media, leading to supramolecular structures, such as micelles [11], liposomes, and bilayers. These supramolecular structures create microenvironments [12], which have been linked to their role as green catalysts in aldol reactions [11, 12], and in bioactive properties such as interactions with biological membranes [4].

As a result of its unique structural properties, bile acids and their synthetic derivatives have emerged as an important class of amphiphilic bioactive molecules with preferential activity against gram‐positive bacteria, highlighting the potential of the bile acid scaffold as a platform for antimicrobial development. For instance, deoxycholic acid (DCA), a secondary bile acid generated by microbial metabolism in the intestine, has been shown to exert a pronounced inhibitory effect on the growth of *E*. *faecalis*. In vitro studies demonstrated that DCA impaired bacterial growth at concentrations as low as 0.01% (100 *μ*g/mL), whereas complete growth inhibition was observed at 0.5% (5000 *μ*g/mL). These findings suggest that DCA contributes to maintaining *E. faecalis* as a subdominant member of the gut microbiota under eubiotic conditions and highlight the antimicrobial potential of hydrophobic bile acids against clinically relevant gram‐positive bacteria [14]. On the other hand, chenodeoxycholic acid (CDCA), a naturally occurring primary bile acid, exhibited MIC values of approximately 320 *μ*g/mL against both methicillin‐susceptible and MRSA, indicating only moderate activity as a standalone antimicrobial agent. Despite these relatively high MIC values, CDCA significantly enhanced the efficacy of aminoglycosides through synergistic interactions, suggesting that its biological relevance may extend beyond direct bacterial growth inhibition [15].

In addition to natural bile acids, structural modifications of bile acids have provided a strategy for synthesizing various substitution patterns to promote antimicrobial applications [16]. Bile acids, particularly cholic acid, have facilitated the synthesis of various derivative families, including dendrimers and oligomers [17, 18], ceragenins [19], and peptide conjugates [7, 8, 20]. These derivatives are aimed at emulating the antimicrobial peptide activity at a lower synthesis cost. Numerous synthetic endeavors have been undertaken to obtain highly efficient bile acid–based compounds against antibiotic‐resistant bacterial strains. Most of the obtained compounds completely substitute Positions 3, 7, 12, and 24 [8, 17, 19, 21], signifying a substantial synthetic effort. Compounds that demonstrate superior performance harbor hydrophobic residues within the system, typically at Position 24, while preserving the hydrophilicity of the *α*‐face through the use of charged amino acids [22]. Previous studies on cancer cell lines indicate that *α*‐amide derivatives exhibit improved activity with increasing log*P* and that a hydroxyl group at carbon 7 (C7) is necessary for compound efficacy [23].

The aforementioned structural modifications result in changes in the physicochemical properties of bile acid derivatives, and, of particular interest, in their lipophilicity. This property has been extremely valuable for screening small molecules with good drug‐like properties [24, 25], but it has also been essential for characterizing the antimicrobial properties of small molecules and peptides [26, 27]. In this context, identifying the optimal hydrophilic–lipophilic balance is central to the rational design of antimicrobial agents to minimize cytotoxicity and other undesired off target effects [9].

By systematically substituting a particular position in the steroid ring, it is possible to study its influence on both the system′s lipophilicity, which affects its aggregation properties and its antimicrobial activity. This allows us to understand the individual impact of each position on these parameters, to identify lead molecules, and therefore efficient synthesis strategies. Synthetic routes based on substitutions at carbon 3*β* have been previously used, yielding products with attractive activity; however, their lipophilicity has not been determined. Thus, incorporating aromatic hydrophobic moieties is a rational strategy to expand the *β*‐face and modulate the hydrophobic surface area. Biphenyl and its derivatives have been used historically as antimicrobial aromatic structural moieties [28, 29] in part due to their exhibited activity in the lipidic membrane [30]. By introducing it at the 3*β* position of the steroid structure, the system′s lipophilicity will increase due to the expansion of the *β*‐face.

In this study, we report the synthesis of five bile acid–derived aminosteroids (AC series) and their corresponding biphenyl‐functionalized derivatives (BIAC series) at the 3*β* position and evaluate their antimicrobial activity against four reference bacterial strains, hemolytic properties, and quantum mechanics–derived lipophilicity (log*P*) values. By incorporating a bioactive biphenyl group into the bile acid scaffold and exploiting structural variations in the steroid nucleus, particularly the number and spatial distribution of hydroxyl groups, we sought to modulate the amphiphilic balance of these molecules. Our objective was to investigate how changes in lipophilicity and molecular topology influence antimicrobial efficacy and cytotoxicity.

## 2. Methods and Materials

### 2.1. Synthesis

The spectral synthesis of the 3‐*β*‐bile acid‐biphenyl derivatives has been previously reported [31].

### 2.2. Spectral Characterization

The spectral characterization of the 3‐*β*‐bile acid‐biphenyl derivatives has been previously reported [31].

### 2.3. Biological Assays

#### 2.3.1. Antibiotic Activity

The strains used were obtained from the American Type Culture Collection (ATCC): *Escherichia coli* ATCC 25922, *Pseudomonas aeruginosa* ATCC 14502, *S*. *aureus* ATCC 6538, and *E*. *faecalis* ATCC 10741. All strains used have no acquired resistance determinants. *S. aureus* ATCC 6538 is a methicillin‐sensitive strain (MSSA). *E. faecalis* ATCC 10741 is a vancomycin‐sensitive strain.

Synthesized compounds were dissolved in DMSO (Sigma‐Aldrich) at a concentration of 2560 *μ*g/mL. Serial dilutions were then performed to 2.5 *μ*g/mL in Mueller–Hinton broth (MHB) (Oxoid) in a 96‐well plate to a final volume of 100 *μ*L. An inoculum of 100 *μ*L of 1 × 10^6^ CFU/mL in MHB for each strain was challenged against 100 *μ*L of the dilutions of the synthesized compounds, resulting in a final concentration of 5 × 10^5^ CFU/mL for each strain and from 1280 to 1.25 *μ*g/mL for each compound. To control bacterial growth and evaluate potential inhibition by DMSO, each bacterial strain was exposed in MHB to serial dilutions of DMSO ranging from 50% to 0.05%. A column was left uninoculated as the negative control. Then, all tubes were incubated at 35°C for 20 h, within 15 min of the experiment setup.

#### 2.3.2. Hemolytic Activity

Hemolytic activity was assessed according to Palermo and Kuroda (CITA). This method is based on the release of hemoglobin from lysed red blood cells (RBCs). Two‐fold dilutions of each compound stock solution were used from 2560 to 10 *μ*g/mL. Freshly collected human RBCs were rinsed three times with phosphate‐buffered saline (PBS) and resuspended in PBS to a final concentration of 3.3% (*v*/*v*). Then, 90 *μ*L of this suspension was mixed with 10 *μ*L of each dilution in a sterile 96‐well plate to a final RBC concentration of 3% and a final compound concentration ranging from 256 to 1 *μ*g/mL. PBS, Triton X‐100, and DMSO were used as negative, positive, and solvent controls for hemolysis. Each dilution and control was performed in triplicate. The plate was incubated in an orbital shaker at 37°C and 250 rpm for 60 min. Then, it was centrifuged at 1000 rpm for 10 min. Ten microliters of the supernatant was diluted into 90 *μ*L of PBS, and the absorbance at 405 nm was observed using a microplate reader.

The hemolysis fraction was defined as the mean absorbance divided by the mean of the positive‐control readings. The absorbance of the negative control was subtracted from the sample absorbance prior to calculation. The hemolysis activity was plotted as a function of substance concentration (*μ*g/mL).

### 2.4. Computational Methods

#### 2.4.1. Log*P* Computations

Conformers of bile acid derivatives were generated using Obabel software from their SMILES notations (see Table S1). The use of Obabel was chosen because the compounds′ rigid nature limited the conformational space available for exploration. Different families of conformers were generated for each compound to account for the orientation of the hydroxyl group′s hydrogen atom. To eliminate redundant conformations, up to six conformers were generated for each molecule and family, and the resulting conformers were visually inspected. A preliminary sampling of the conformational preferences of the compounds was performed by optimizing the geometries of the generated conformers in water and n‐octanol. This was carried out using the B3LYP/6‐31G(d) level of theory with the SMD model. We have calculated the thermal corrections to estimate the free energy of conformations in water and *n*‐octanol [32]. Single‐point energy calculations in the gas phase were performed to estimate the solvation‐free energy of each conformation. The log*P* was determined from the Boltzmann‐weighted population of conformational families obtained in water and *n*‐octanol. All calculations were performed using Gaussian 09 [32]. For comparison purposes, log*P* values were also calculated using the XlogP tool which is an atom fragment‐based method that estimates the octanol/water partition coefficient by summing the lipophilic contributions of individual atoms and applying structural correction factors [33] and using ACD/LogP commercial software using a fragment‐based approach combined with proprietary correction factors derived from large experimental datasets.

## 3. Results

### 3.1. 3‐b‐Aminosteroids and 3‐Amine‐Modified Steroids

Table 1 shows the structure of bile acids derivatives which were synthetically modified to obtain different substitution patterns.

**Table 1:** Structure of bile acids derivatives.

Bile acid parent	Structure	R =	
		H	
Cholic acid		AC01C	BIAC01C
Deoxycholic acid		AC02D	BIAC02D
Ursodeoxycholic acid		AC04U	BIAC04U
Chenodeoxycholic acid		AC05Q	BIAC05Q
Lithocholic acid		AC03L	BIAC03L

**Figure 2:**
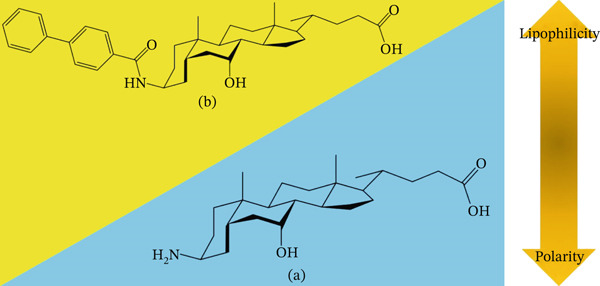
(a) Structure of chenodeoxycholic‐3b‐aminosteroids (AC05Q) and (b) their modified analogs with the biphenyl moiety (BIAC05Q).

In order to do a systematic study on lipophilicity, we chose to functionalize the C3 position with an amino group, which changes the orientation of the group to the *β*‐face. As shown in Table 1 and Figure 2, this modification allows obtaining two molecular scaffolds, the amino and the biphenyl form. Although the amino form is hydrophilic, the biphenyl form will extend the *β*‐face, leading to an increase in the lipophilicity. The main motivation for adding the biphenyl moiety is twofold: to enhance the compounds′ lipophilicity and thereby improve their physicochemical properties [30, 34] and to provide them with an amphipathic and bioactive character, which is widely recognized to be related to antimicrobial activity [35, 36].

### 3.2. Structural and Lipophilicity Analysis

The calculated lipophilicity values revealed a marked difference between the parent aminosteroids (AC series) and their biphenyl‐substituted analogs (BIAC series). For the AC compounds, QM‐log*P* predicted strongly negative values, ranging from −5.83 to −3.90, indicating a predominantly hydrophilic character. This behavior can be attributed to the zwitterionic nature of these molecules, which was explicitly considered in the quantum mechanical calculations. In contrast, the fragment‐based methods XlogP and ACD/LogP predicted positive log*P* values for the same compounds, reflecting their limited ability to fully account for charge separation and solvation effects associated with zwitterionic species. These results suggest that QM‐log*P* provides a more realistic description of the physicochemical behavior of the parent aminosteroids in aqueous environments where ionic compounds are predominant as the chemoinformatic LiProS tool predict [37].

The introduction of the biphenyl moiety dramatically increased lipophilicity across the BIAC series (see Figure 2). All three methods consistently predicted a substantial shift toward hydrophobicity, with QM‐log*P* values increasing to 4.53–7.61. This trend is consistent with the incorporation of a highly hydrophobic aromatic fragment into the bile acid scaffold and supports the rationale of using the biphenyl group to modulate amphiphilic balance. Notably, the BIAC derivatives retained the expected dependence of lipophilicity on the hydroxylation pattern of the steroid nucleus, with the nonhydroxylated derivative BIAC03L displaying the highest lipophilicity, followed by the monohydroxylated (BIAC02D, BIAC04U, and BIAC05Q) and dihydroxylated analog (BIAC01C).

An important observation is that QM‐log*P* was the only method capable of clearly discriminating among the monohydroxylated compounds and resolving subtle differences within the BIAC series. Although XlogP and ACD/LogP assigned identical values to several derivatives (e.g., BIAC02D, BIAC04U, and BIAC05Q), the QM‐based approach captured variations arising from the position and orientation of hydroxyl groups on the steroid framework. These seemingly small differences in lipophilicity may be biologically relevant, as membrane interactions, cellular uptake, aggregation behavior, and selectivity are often highly sensitive to fine changes in molecular polarity. Consequently, the enhanced resolution provided by QM‐log*P* may be particularly valuable when interpreting biological assays and establishing structure–activity relationships [38, 39], where subtle physicochemical differences can translate into significant variations in antimicrobial activity and toxicity.

### 3.3. Biological Activity

#### 3.3.1. Antibiotic Activity

Table 2 shows that all tested bile acid derivatives showed no inhibitory effect on gram‐negative bacteria at 160 *μ*g/mL. The lack of activity against gram‐negative bacteria has been reported and aligns with the established understanding that the outer membrane acts as a permeability barrier, restricting the interaction with antimicrobial compounds [40]. Regarding gram‐positive bacteria, two compounds exert effects in both *S. aureus* and *E. faecalis* models. BIAC03L had MICs of 10 *μ*g/mL in both bacterial models, whereas BIAC05Q had an MIC of 80 *μ*g/mL. Notably, the core molecules lacking biphenyl (AC series) do not affect the same bacterial models. Thus, the augmented inhibitory activity is attributable, at least in part, to this lipophilic modification.

**Table 2:** Minimal inhibitory concentrations (MIC) of synthesized compounds against tested strains.

Compound	*E. coli* ATCC 25,922 *μ*g/mL (mmo/L)	*P. aeruginosa* ATCC 14,502 *μ*g/mL (mmol/L)	*S. aureus* ATCC 6538 *μ*g/mL (mmol/L)	*E. faecalis* ATCC 10,741 *μ*g/mL (mmol/L)
BIAC01C	160 (0.272)	160 (0.272)	160 (0.272)	160 (0.272)
BIAC02D	160 (0.280)	160 (0.280)	160 (0.280)	160 (0.280)
BIAC03L	160 (0.288)	160 (0.288)	**10 (0.018)**	**10 (0.018)**
BIAC04U	160 (0.280)	160 (0.280)	160 (0.280)	160 (0.280)
BIAC05Q	160 (0.280)	160 (0.280)	**80 (0.140)**	**80 (0.140)**
AC01C	160 (0.393)	160 (0.393)	160 (0.393)	160 (0.393)
AC02D	160 (0.409)	160 (0.409)	160 (0.409)	160 (0.409)
AC03L	160 (0.426)	160 (0.426)	160 (0.426)	160 (0.426)
AC04U	160 (0.409)	160 (0.409)	160 (0.409)	160 (0.409)
AC05Q	160 (0.409)	160 (0.409)	160 (0.409)	160 (0.409)

*Note:* The values highlighted in bold indicate the results that showed promising activity.

#### 3.3.2. Hemolytic Activity

**Figure 3:**
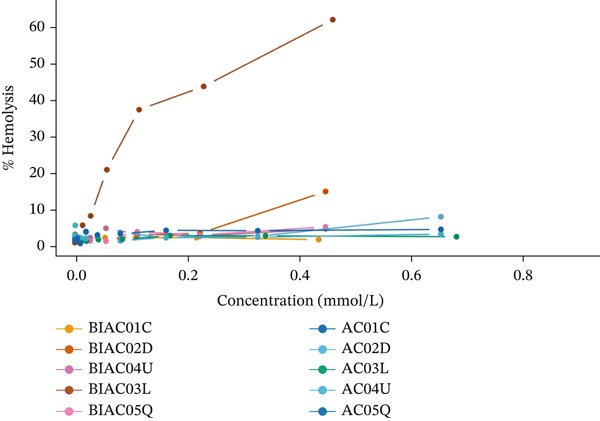
Hemolytic activity (as a percentage) at different concentrations of bile salt derivatives was measured.

Amphipathicity is closely associated with the bioactivity of compounds against pathogens; however, it must be modulated to avoid the formation of highly toxic, poorly selective compounds [9]. Figure 3 shows that BIAC03L (nonhydroxylated steroid derivative) is the most active compound with respect to hemolytic activity in the biphenyl‐substituted series. Monohydroxylated (BIAC02D, BIAC04U, and BIAC05Q), dihydroxylated analogs (BIAC01C), and AC series molecules exhibit minimal hemolytic activity.

## 4. Discussion

Lipophilicity is widely recognized as one of the most important physicochemical properties governing the biological performance of bioactive molecules [41]. Consequently, the primary objective of this study was to modulate the lipophilicity of bile acid–derived aminosteroids through the introduction of a biphenyl moiety and variations in the hydroxylation pattern of the steroid scaffold. In this study, a dataset including 10 bile acid derivatives is used, the AC series including 3‐amino steroid (log*P* < 0, see Table 3) and BIAC derivatives prepared with biphenyl (log*P* > 0, see Table 3). As expected, the nonhydroxylated steroid derivatives 3‐aminolithocholic acid (AC03L) and BIAC03L stand out for their high lipophilicity within their respective groups.

**Table 3:** Calculated *n*‐octanol/water partition coefficient (log*P*) for the aminosteroids and their modified analogs with the biphenyl moiety using quantum mechanics–derived lipophilicity, QM‐log*P*, free fragment‐based XlogP, and commercial fragment‐based ACD/logP.

Compound	QM‐log*P*	XlogP	ACD/LogP
AC01C^a^	−5.83	0.93	−0.62
AC02D^a^	−4.66	2.41	0.69
AC03L^a^	−3.90	3.33	2.06
AC04U^a^	−5,46	2.41	0.69
AC05Q^a^	−5.30	2.41	0.69
BIAC01C	4.53	5.30	5.03
BIAC02D	6.19	6.42	6.78
BIAC03L	7.61	7.71	8.12
BIAC04U	6.30	6.42	6.78
BIAC05Q	6.07	6.42	6.78

^a^For these compounds, the zwitterionic structure was used in the calculations.

**Figure 4:**
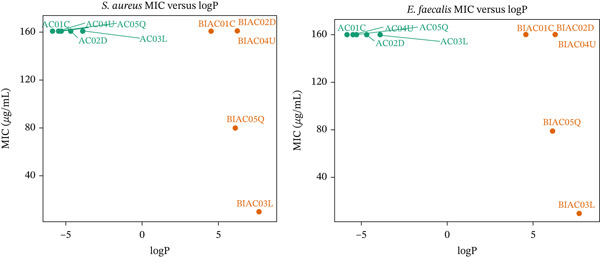
Minimal inhibitory concentration (MIC) of the derivatives and their relationship with the QM‐based log*P*.

The relationship between MIC and QM‐log*P* (see Figure 4) revealed a clear separation between the parent aminosteroids (AC series) and the biphenyl‐substituted derivatives (BIAC series). The AC compounds clustered within the hydrophilic region (negative QM‐log*P* values) and displayed relatively weak antibacterial activity, with MIC values of approximately 160 *μ*g/mL against both *S. aureus* and *E. faecalis*. In contrast, the BIAC derivatives formed a distinct, more lipophilic group and generally exhibited enhanced antimicrobial activity. Notably, BIAC03L, the most lipophilic compound in the series (QM − log*P* = 7.61), showed the lowest MIC values against both bacterial species, whereas the less lipophilic BIAC derivatives displayed intermediate activities. These results suggest that increasing lipophilicity through biphenyl incorporation improves antibacterial potency, likely by enhancing interactions with bacterial membranes. Furthermore, the ability of QM‐log*P* to clearly separate the compounds into hydrophilic and hydrophobic groups highlights its value for interpreting structure–activity relationships and identifying subtle physicochemical factors underlying the observed biological responses. These trends compare well with previous work on gram‐positive bacteria, *S. aureus*, in which bile acid derivatives reported MIC values ranging from 4 to 128 *μ*g/mL [5–8, 18, 20, 42–44].

**Figure 5:**
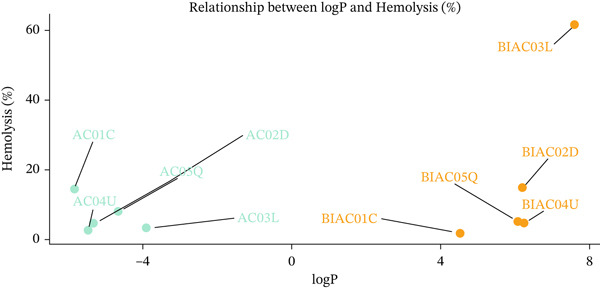
Hemolytic activity (as a percentage) at 256‐*μ*g/mL concentration of the derivatives and their relationship with the QM‐based log*P*.

Because the relationship between hemolytic activity and QM‐log*P* (see Figure 5) highlights the importance of considering toxicity alongside antimicrobial potency when evaluating these bile acid derivatives. As observed for the MIC data, the QM‐log*P* values clearly separate the compounds into two groups, with the hydrophilic AC derivatives exhibiting negative log*P* values and the more lipophilic BIAC derivatives displaying positive log*P* values. However, increased lipophilicity did not translate uniformly into higher hemolytic activity. Although BIAC03L was the most active compound against both *S. aureus* and *E. faecalis*, it also exhibited the highest hemolysis (c.a 62%), indicating a reduced degree of selectivity toward bacterial membranes over mammalian cell membranes. This finding underscores that antimicrobial activity alone is insufficient to identify the most promising lead compounds. In contrast, BIAC05Q maintained enhanced antibacterial activity relative to the parent aminosteroids while displaying substantially lower hemolysis (5%), comparable with that of several less lipophilic derivatives. Therefore, BIAC05Q emerges as a particularly attractive candidate, achieving a more favorable balance between antimicrobial efficacy and toxicity.

The enhanced antimicrobial activity observed for the BIAC derivatives may not arise exclusively from the properties of individual molecules but also from their ability to form supramolecular assemblies in aqueous environments [11, 12]. Bile acids are facially amphiphilic molecules that possess a well‐documented tendency to self‐associate into dimers, oligomers, and larger aggregates through hydrophobic interactions between their steroidal faces. The introduction of a biphenyl moiety further increases molecular hydrophobicity and may promote aggregation phenomena, potentially generating bioactive supramolecular species rather than isolated molecular entities.

This possibility was particularly relevant for the BIAC series, where increased lipophilicity was accompanied by enhanced antimicrobial activity. Mechanistically, the antimicrobial effects of bile acid–based systems appear to extend beyond simple membrane permeabilization. The supramolecular organization in bile acid is linked with the antimicrobial activity; thus, noncovalent oligomers formed through the self‐assembly of bile acid derivatives have been reported to exhibit potent antibacterial properties, indicating that aggregation can enhance the facial amphiphilicity required for selective interactions with bacterial membranes. In such systems, the aggregate itself, rather than the individual monomer, behaves as the biologically active species. This concept is consistent with the behavior of many amphiphilic antimicrobials, where nanoscale assemblies concentrate hydrophobic and hydrophilic domains into architectures that interact more efficiently with biological membranes. In addition, bile acid–derived compounds can induce widespread protein unfolding, protein aggregation, and disulfide stress within bacterial cells, revealing that bile acid toxicity involves profound disruption of intracellular proteostasis [45, 46].

Within this framework, the superior activity of BIAC03L may be interpreted as an enhanced propensity to self‐associate. The monohydroxylated lithocholic acid scaffold provides the highest QM‐*logP* value and the lowest antimicrobial MICs, suggesting that subtle changes in hydroxylation pattern influence the balance between solubility and aggregation. This interpretation is supported by the aggregation behavior of the parent bile acids, as lithocholic acid exhibits the lowest critical micelle concentration (CMC ≈1 mM, see Table S2) among the naturally occurring bile acids, indicating a strong tendency to form supramolecular assemblies. Such a high propensity for self‐association may facilitate the formation of bioactive aggregates capable of interacting efficiently with bacterial membranes, thereby enhancing antimicrobial activity. However, these same physicochemical properties may also explain the elevated hemolytic activity observed for BIAC03L, as aggregates that effectively perturb bacterial membranes may similarly disrupt erythrocyte membranes.

In contrast, BIAC05Q, derived from CDCA, displayed a more favorable balance between antimicrobial activity and hemocompatibility. CDCA possesses a substantially higher CMC (4–9 mM, see Table S2) than lithocholic acid, reflecting a lower tendency to self‐aggregate. Consequently, the corresponding biphenyl derivative may form supramolecular structures that are sufficiently stable to promote antimicrobial activity while avoiding the excessive membrane‐disruptive behavior associated with BIAC03L. The lower hemolytic activity of BIAC05Q, together with its retained antibacterial potency, suggests that optimal biological performance may be achieved not by maximizing lipophilicity or aggregation propensity, but by fine‐tuning the self‐assembly behavior of the amphiphilic system.

Therefore, the present results support the hypothesis that aggregation‐prone bile acid derivatives constitute a promising class of antimicrobial agents and that their biological activity should be considered from a supramolecular perspective, where self‐assembled structures rather than individual molecules may represent the true bioactive species. The contrasting behaviors of the lithocholic acid–derived BIAC03L and the CDCA‐derived BIAC05Q further suggest that the degree of aggregation, governed by subtle structural variations in the bile acid scaffold, may play a central role in determining the balance between antimicrobial efficacy and toxicity. Importantly, this interpretation was only evident when lipophilicity was evaluated using the QM‐log*P* approach. Unlike the fragment‐based methods, QM‐log*P* successfully distinguished the subtle lipophilicity differences among the nonhydroxylated, monohydroxylated, and dihydroxylated, capturing the influence of hydroxyl group number and position on the physicochemical properties of the molecules.

**Figure 6:**
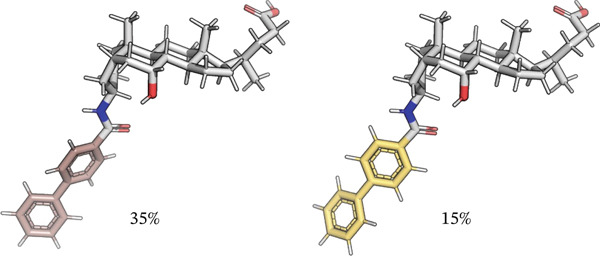
Most contributing conformations in water of compound BIAC05Q. Biphenyl groups in red and yellow represent the main difference between the two conformations. Low‐energy conformations are relevant to define the spatial distribution of polar and nonpolar regions within the molecules determining their amphiphilic character.

Another factor influencing the activity of bile acid derivatives is the presence and orientation of a hydroxyl group at C7 of the steroid nucleus. BIAC05Q, derived from CDCA, exhibited higher antimicrobial activity than its epimer BIAC04U, derived from ursodeoxycholic acid. These results indicate that not only the presence but also the stereochemical orientation of the hydroxyl group at C7 plays an important role in determining the biological activity of bile acid derivatives. In aqueous solution, bile acids adopt conformations that expose a hydrophilic and a hydrophobic face; in BIAC05Q, the OH in C7 and the polarizable biphenyl substituent strengthen the polar side of the compound (see Figure 6), and subtle changes in hydroxyl group orientation can significantly affect this facial amphiphilicity. The axial 7*α*‐hydroxyl group of BIAC05Q is expected to enhance the segregation of polar and nonpolar regions within the steroid scaffold, resulting in a more pronounced amphiphilic character than the equatorial 7*β*‐hydroxyl group present in BIAC04U. Similarly, BIAC02D, in which the second hydroxyl group is located at C12, displays a different distribution of polarity across the steroid nucleus, further altering its amphiphilic balance.

This interpretation is supported by the QM‐log*P* calculations, which were uniquely able to resolve these subtle physicochemical differences. Among the dihydroxylated derivatives, BIAC05Q displayed the lowest QM‐log*P* value, indicating a distinct balance between hydrophilicity and hydrophobicity that was not captured by the fragment‐based methods. Interestingly, the parent bile acid of BIAC05Q, CDCA, exhibits an intermediate critical micelle concentration (CMC = 4–9 mM, see Table S2), compared with the lower CMC of DCA (2–10 mM, typically lower on average) and the broader range reported for ursodeoxycholic acid (7–19 mM, see Table S2). This intermediate aggregation propensity may favor the formation of supramolecular assemblies that are sufficiently stable to promote interactions with bacterial membranes while avoiding the excessive membrane disruption associated with higher hemolytic activity. Consistent with this hypothesis, BIAC05Q showed a more favorable balance between antimicrobial efficacy and hemocompatibility than either BIAC03L or the other dihydroxylated derivatives. Collectively, these findings suggest that subtle structural variations affecting amphiphilicity, self‐association, and aggregate formation can strongly influence biological activity, supporting the view that the supramolecular behavior of bile acid derivatives is a key determinant of their antimicrobial and toxicological profiles.

## 5. Conclusion

The present study demonstrates that rational modulation of lipophilicity through biphenyl functionalization of bile acid–derived aminosteroids is an effective strategy for enhancing antimicrobial activity. Among the methods evaluated, QM‐log*P* proved particularly valuable because it captured subtle differences in amphiphilicity that were not resolved by conventional fragment‐based approaches. This enhanced sensitivity allowed discrimination among derivatives differing only in the number, position, and orientation of hydroxyl groups, providing insights into how structural modifications influence the balance between hydrophilic and hydrophobic domains. The marked increase in antimicrobial activity observed for the BIAC series relative to the parent AC compounds suggests that increasing amphiphilicity promotes more favorable interactions with bacterial membranes and may also enhance the propensity of these molecules to form supramolecular assemblies. Taken together with literature reports describing aggregation‐dependent biological activity in bile acid systems, the results support the hypothesis that self‐assembled structures, rather than individual molecules, may constitute the bioactive species responsible for the improved antimicrobial performance of the BIAC derivatives.

Importantly, the biological evaluation revealed that antimicrobial potency alone is insufficient to identify the most promising lead compound. Although BIAC03L exhibited the lowest MIC values, it also showed the highest hemolytic activity, indicating a reduced degree of selectivity. In contrast, BIAC05Q achieved a more favorable balance between antibacterial activity and hemocompatibility, emerging as the most promising derivative of the series. This behavior appears to be associated with the structural characteristics of the CDCA scaffold, particularly the presence of the axial hydroxyl group at C7, which enhances facial amphiphilicity while maintaining a balanced aggregation propensity. Notably, BIAC05Q exhibited substantially greater antibacterial activity than its parent bile acid and surpassed activities reported for CDCA against *S*. *aureus* (MIC ≈320 *μ*g/mL) and for DCA against *E*. *faecalis*, where complete growth inhibition required concentrations as high as 5000 *μ*g/mL. These findings highlight the importance of fine‐tuning amphiphilicity and supramolecular behavior through precise structural modifications and establish QM‐log*P*–guided design as a powerful approach for the development of bile acid–based antimicrobial agents with improved efficacy and selectivity.

## Author Contributions

A.M.A.: methodology, validation, data curation, writing—original draft, and writing—review and editing. L.R‐M.: methodology, validation, data curation, writing—original draft, and writing—review and editing. W.J.Z.: conceptualization, methodology, validation, data curation, writing—original draft, and writing—review and editing. K.B.: methodology and validation. V.H.S‐T.: conceptualization, methodology, validation, data curation, writing—review and editing, and funding acquisition.

## Funding

This study was supported by the School of Chemistry and the Center for Electrochemistry and Chemical Energy (CELEQ) of the Universidad de Costa Rica.

## Conflicts of Interest

The authors declare no conflicts of interest.

## Supporting information


**Supporting Information** Additional supporting information can be found online in the Supporting Information section. The Supporting Information contains additional experimental data supporting the results presented in this study. Table S1: The smiles notations of bile acids derivatives. Table S2: The CMC data of bile acids and their derivatives taken from the literature.

## Data Availability

The data that support the findings of this study are available from the corresponding authors upon reasonable request.
