# Supplementary material for: Impact of Lipophilicity‐Tuning on the Antimicrobial Activity of a Series of β‐Face–Expanding Bile Acid Derivatives
Source: Biomed Res Int. 2026 Jul 30;2026:5550616. doi: 10.1155/bmri/5550616 (PMC13424823; doi:10.1155/bmri/5550616)
Supplement: Supplementary file 1 — Supporting Information Additional supporting information can be found online in the Supporting Information section. The Supporting Information contains additional experimental data supporting the results presented in this study. Table S1: The smiles notations of bile acids derivatives. Table S2: The CMC data of bile acids and their derivatives taken from the literature. [file BMRI-2026-5550616-s001.docx]

**Supplementary Material**

**Impact of Lipophilicity-Tuning on the Antimicrobial Activity of a New Series of β-Face-Expanding Bile Acid Derivatives**

Allan Mora Abarca^a^; Luis Rivera Montero^b^; Kenia Barrantes^b^; Victor H. Soto-Tellini^a^*, William J. Zamora^c,d,e^*

^a^Center for Research in Electrochemistry and Chemical Energy (CELEQ), University of Costa Rica, 11501-2060, Costa Rica.

^b^ Health Research Institute, University of Costa Rica, , San José, 11501-2060, Costa Rica

^c^CBio3 Laboratory, School of Chemistry, University of Costa Rica, San José, 11501-2060, Costa Rica.

^d^Laboratory of Computational Toxicology and Artificial Intelligence (LaToxCIA), Biological Testing Laboratory (LEBi), University of Costa Rica, San Pedro, San José, Costa Rica.

^e^National Advanced Computing Collaboratory (CNCA), National High Technology Center (CeNAT), Costa Rica.

The SMILES notations and Critical Micelle Concentration (CMC) are found in the following sections.

**Table S1.** Smiles notations of bile acids derivatives………………………………………………….3

**Table S2.** CMC data of bile acids and their oxoderivatives taken from the literature………………4

[**References** 4](#_Toc231989283)

**Table S1.** Smiles notations of bile acids derivatives.

| ID | SMILES |
| --- | --- |
| BIAC01C | CC(CCC(O)=O)C1[C@]2(C)[C@@H](CC1)[C@H]3[C@H](C[C@@H]2O)[C@]4(C)[C@H](C[C@H]3O)C[C@@H](NC(C5=CC=C(C6=CC=CC=C6)C=C5)=O)CC4 |
| BIAC02D | CC(CCC(O)=O)C1[C@]2(C)[C@@H](CC1)[C@H]3[C@H](C[C@@H]2O)[C@]4(C)[C@H](CC3)C[C@@H](NC(C5=CC=C(C6=CC=CC=C6)C=C5)=O)CC4 |
| BIAC03L | CC(CCC(O)=O)C1[C@]2(C)[C@@H](CC1)[C@H]3[C@H](CC2)[C@]4(C)[C@H](CC3)C[C@@H](NC(C5=CC=C(C6=CC=CC=C6)C=C5)=O)CC4 |
| BIAC04U | CC(CCC(O)=O)C1[C@]2(C)[C@@H](CC1)[C@H]3[C@H](CC2)[C@]4(C)[C@H](C[C@@H]3O)C[C@@H](NC(C5=CC=C(C6=CC=CC=C6)C=C5)=O)CC4 |
| BIAC05Q | CC(CCC(O)=O)C1[C@]2(C)[C@@H](CC1)[C@H]3[C@H](CC2)[C@]4(C)[C@H](C[C@H]3O)C[C@@H](NC(C5=CC=C(C6=CC=CC=C6)C=C5)=O)CC4 |
| AC01C | CC(CCC([O-])=O)C1[C@]2(C)[C@@H](CC1)[C@H]3[C@H](C[C@@H]2O)[C@]4(C)[C@H](C[C@H]3O)C[C@@H]([NH3+])CC4 |
| AC02D | CC(CCC([O-])=O)C1[C@]2(C)[C@@H](CC1)[C@H]3[C@H](C[C@@H]2O)[C@]4(C)[C@H](CC3)C[C@@H]([NH3+])CC4 |
| AC03L | CC(CCC([O-])=O)C1[C@]2(C)[C@@H](CC1)[C@H]3[C@H](CC2)[C@]4(C)[C@H](CC3)C[C@@H]([NH3+])CC4 |
| AC04U | CC(CCC([O-])=O)C1[C@]2(C)[C@@H](CC1)[C@H]3[C@H](CC2)[C@]4(C)[C@H](C[C@@H]3O)C[C@@H]([NH3+])CC4 |
| AC05Q | CC(CCC([O-])=O)C1[C@]2(C)[C@@H](CC1)[C@H]3[C@H](CC2)[C@]4(C)[C@H](C[C@H]3O)C[C@@H]([NH3+])CC4 |

**Table S2.** CMC data of bile acids and their oxoderivatives taken from the literature [1].

| **Bile acids** | **CMC (mmol/L), 25^∘^C** |
| --- | --- |
| cholic acid | 6-18 |
| deoxycholic acid | 2-10 |
| lithocholic acid | 1 |
| ursodeoxycholic acid | 7-19 |
| chenodeoxycholic acid | 4-9 |

# **References**

1. Szekeres, M., Viskolcz, B., Poša, M., Csanádi, J., Škorić, D., Illés, E., Tóth, I. Y., & Tombácz, E. (2014). The effect of hydroxyl moieties and their oxosubstitution on bile acid association studied in floating monolayers. *TheScientificWorldJournal*, *2014*, 152972. https://doi.org/10.1155/2014/152972
